# Active case detection in national visceral leishmaniasis elimination programs in Bangladesh, India, and Nepal: feasibility, performance and costs

**DOI:** 10.1186/1471-2458-12-1001

**Published:** 2012-11-20

**Authors:** M Mamun Huda, Siddhivinayak Hirve, Niyamat Ali Siddiqui, Paritosh Malaviya, Megha Raj Banjara, Pradeep Das, Sangeeta Kansal, Chitra Kumar Gurung, Eva Naznin, Suman Rijal, Byron Arana, Axel Kroeger, Dinesh Mondal

**Affiliations:** 1 Centre for Communicable Diseases, icddr,b, Dhaka, Bangladesh; 2 KEM Hospital Research Center, Pune, India; 3 Umea Center for Global Health Research, Umea University, Umea, Sweden; 4 Rajendra Memorial Research Institute of Medical Sciences, Patna, India; 5 Institute of Medical Sciences, Benares Hindu University, Varanasi, India; 6 Tribhuvan University, Kathmandu, Nepal; 7 Consultant, World Health Organization, Geneva, Switzerland; 8 BP Koirala Institute of Health Sciences, Dharan, Nepal; 9 World Health Organization, Special Program for Research and Training in Tropical Diseases, Geneva, Switzerland; 10 Liverpool School of Tropical Medicine, Liverpool, UK; 11 Centre for Nutrition and Food Security, icddr,b, Dhaka, Bangladesh

**Keywords:** Visceral leishmaniasis, Kala-azar, Post kala-azar dermal leishmaniasis, Active case detection, Elimination progamme, Bangladesh, India, Nepal

## Abstract

**Background:**

Active case detection (ACD) significantly contributes to early detection and treatment of visceral leishmaniasis (VL) and post kala-azar dermal leishmaniasis (PKDL) cases and is cost effective. This paper evaluates the performance and feasibility of adapting ACD strategies into national programs for VL elimination in Bangladesh, India and Nepal.

**Methods:**

The camp search and index case search strategies were piloted in 2010-11 by national programs in high and moderate endemic districts / sub-districts respectively. Researchers independently assessed the performance and feasibility of these strategies through direct observation of activities and review of records. Program costs were estimated using an ingredients costing method.

**Results:**

Altogether 48 camps (Bangladesh-27, India-19, Nepal-2) and 81 index case searches (India-36, Nepal-45) were conducted by the health services across 50 health center areas (Bangladesh-4 Upazillas, India-9 PHCs, Nepal-37 VDCs). The mean number of new case detected per camp was 1.3 and it varied from 0.32 in India to 2.0 in Bangladesh. The cost (excluding training costs) of detecting one new VL case per camp varied from USD 22 in Bangladesh, USD 199 in Nepal to USD 320 in India. The camp search strategy detected a substantive number of new PKDL cases. The major challenges faced by the programs were inadequate preparation, time and resources spent on promoting camp awareness through IEC activities in the community. Incorrectly diagnosed splenic enlargement at camps probably due to poor clinical examination skills resulted in a high proportion of patients being subjected to rK39 testing.

**Conclusion:**

National programs can adapt ACD strategies for detection of new VL/PKDL cases. However adequate time and resources are required for training, planning and strengthening referral services to overcome challenges faced by the programs in conducting ACD.

## Background

The global burden estimate for Visceral leishmaniasis (VL) also known as kala-azar in the Indian sub-continent, is 0.5 and 2.5 million incident and prevalent cases per year, respectively [1]. With an estimated 200 million people at risk, India, Nepal and Bangladesh harbour an estimated 67% of the global VL disease burden [2]. In this region, *Leishmania donovani* is the only species causing VL, the female sand fly *Phlebotomas argentipes* is the only vector and humans are the only known reservoir [3-5]. These unique characteristics, recent advances in rapid field based diagnostics and availability of effective and safe oral drugs make the disease a target for elimination (reducing incidence to less than one per 10,000 population by 2015) [6]. National programs for kala-azar elimination (NKEP) were launched in 2005 by Bangladesh, India and Nepal. One of the main strategies is early case detection and management of VL and Post Kala-azar Dermal Leishmaniasis (PKDL) [7]. Campaigns were recently undertaken by the NKEP in Bangladesh. In India, fortnight-long campaigns are carried out annually to raise VL awareness in endemic districts. The NKEP in Nepal has strategies of active case detection (ACD) including camp approach and index case approach. The District / Upazilla (Sub-district) level health workers have been trained in ACD in the program. The World Health Organization, Special program for Tropical Diseases Research (TDR) is supporting operational research in these three countries in consultation with national and state VL elimination programs. VL incidence in 2006-07 ranged between 9 (in Nepal) to about 30 per 10,000 population in endemic districts of India and Bangladesh [8]. Though community awareness of VL is high (except in Bangladesh), diagnosis is delayed by several weeks, treatment interruption is frequent, compliance is low, and health provider’s knowledge about VL is limited. ACD significantly contributes to detecting more VL and PKDL cases, early diagnosis and early onset of treatment and lower costs for patients [9]. The need for ACD is enhanced by the advent of a new form of treatment, liposomal Amphotericine B (Ambisome, ™) which is highly effective and can be implemented in small hospitals [10]. A comparison of cost-effectiveness of four different strategies for ACD including Camp strategy (fever camps with spleen examination and rapid diagnostic test (rK39)), Index case (focal) strategy (house to house screening in the neighborhood of recent VL cases), Incentive based strategy (case detection through village health workers who receive an incentive for every new case) and house to house screening strategy (as reference approach) shows 80% sensitivity for the camp approach with high cost-effectiveness. The Index case approach is also cost effective but has lower sensitivity (only few new cases detected in the neighborhood of known VL cases). The Incentive based approach shows high cost-effectiveness in India and Nepal but is not practical in Bangladesh [11]. NKEPs need the right combination of different ACD strategies based on the epidemiological profile, affordability and organizational feasibility.

The Kala-azar Regional Technical Advisory Group (RTAG) recommends the adoption of ACD strategies into NKEPs through the existing health services at sub-district level [12]. In this paper, we assess the performance and feasibility of two known cost-effective ACD strategies i.e. camp and index case search as implemented by the health services, and provide evidence for policies to improve VL case detection in national programs.

## Methods

### Ethics statement

The study was approved by the Ethics Committees of all participating institutes (Ethics Committee, Rajendra Memorial Research Institute of Medical Sciences, India; Ethics Committee, Institute of Medical Sciences, Banaras Hindu University, India; Institutional Review Board, Institute of Medicine, Tribhuvan University, Nepal; Ethics Review Committee, icddr,b, Bangladesh; and Ethics Review Committee, World Health Organization). Subjects participated in the research after a written informed consent.

### Study area and study design

Known VL endemic Upazillas/Primary health centers (PHCs) in Bangladesh and India were categorized into high, moderate and low endemicity. As per national guideline in Bangladesh the high, moderate and low endemicity is defined as VL incidence >2.5, >1.1 to ≤2.5, and <1 per 10,000 population respectively at Upazilla level. In India, VL incidence was calculated based on past one year case load at PHC level and the level of endemicity was defined as high (Incidence >4 per 10,000 population), moderate (1 to 4 per 10,000) and low (< 1 per 10,000). The camp strategy was conducted in areas with high VL endemicity and the index case strategy in areas with moderate VL endemicity. In Nepal with relatively low VL incidence, according to the national program guidelines, index case search was carried out in villages/toles which reported a new VL case in the last one year while villages which reported higher number of VL cases were selected for the camp search strategy. ACD was conducted by the existing health system and its performance was evaluated independently by the research teams in the study area.

### Pre-intervention

As a first step, the Kala-azar RTAG comprising of international and national experts and country program managers from Bangladesh, India and Nepal were presented with country specific findings and recommendations of recent operational research undertaken by the authors. RTAG in turn urged member states to adapt these recommendations into their country programs.

A VL case was defined following national guidelines as a person from an endemic area with fever for more than 2 weeks, splenomegaly and a positive rK39 test. A PKDL case was defined following national guidelines as a person with past history of VL presenting with maculo-papular or nodular skin lesions and a positive rK39 test. Detailed standard operating procedures (SOPs) for camp and index case search strategies were developed in consultation with national and state level program managers for standardizing the interventions as much as possible. After extensive consultations, the countries decided to pilot these interventions at district / sub-district level. Training materials and courses were developed and adapted in the local language for district and health center level functionaries as well as community based health volunteers.

### Intervention

The camp search and the index case search strategy were implemented by the health services in the period March - April, 2010 in Bangladesh and July 2010 - April, 2011 in India and Nepal. The index case search strategy was not implemented in Bangladesh as it was yet to be adopted by the national health authorities.

Camps were scheduled 15-30 days in advance under the aegis of the medical officer of the health center. In India and Nepal, camps were held in the main center of the village and in Bangladesh also in the EPI centers or primary schools. Camps were promoted 1 to 2 days before by health workers and community based health volunteers through house to house visits and public address systems (miking) inviting people with fever more than 2 weeks or known past cases of VL with skin lesions to get examined at the camp. Photographs of skin lesions were displayed to create awareness about PKDL. Camps were conducted by a team of 4 to 10 members comprising of medical officer, laboratory technician, health supervisor, health workers and community based health volunteers like accredited social health activists and anganwadi workers in India. Patients with fever for more than 2 weeks were examined for spleen enlargement by the medical officer and rK39 tests (Kala-azar Detect from InBios International, USA) performed if VL was suspected. A known past case of VL with PKDL-like skin lesions was tested for rK39 test. VL and PKDL cases identified were referred to the local health centers or hospitals for initiation and monitoring of treatment. A roster of camp attendees, case referral forms and records of newly detected VL / PKDL patients was maintained at the health center.

An index case was defined as a recently (within last 12 months) diagnosed or current VL case. A list of index cases was made and index cases were traced at their homes by the health staff. All households within a 50-75 m radius (about 100 households) of the index case household were screened by health workers. Any household member identified with fever for more than 2 weeks or any known VL case with suspected skin lesions was referred to the health center for spleen examination and rK39 testing and management. Record of newly diagnosed VL/PKDL patients was maintained at the health center.

### Evaluation of intervention

The intervention was independently evaluated by researchers while it was ongoing. In India, 4 sub-centers each were randomly selected for evaluation from 7 high and 2 moderate endemic PHCs. In Nepal, 2 high and 35 moderately endemic Village development councils (VDCs) were randomly selected while in Bangladesh which implemented only the camp search strategy, 4 high endemic Upazillas were selected for evaluation. In India and Nepal, the performance of the camp and index case search was assessed by the yield of newly diagnosed VL / PKDL cases, as well as through structured observations by researchers present before, during and following camp or index case search activities. Activities done as per national guidelines and SOPs were defined as adequate or inadequate if otherwise. In Bangladesh, due to late release of funds by the government, camps were scheduled quickly by the program managers and the researchers came to know about the camps only after they were held. As a result, in Bangladesh the camp search strategy could be assessed only in retrospect based on interviews of medical officers who had conducted the camps and review of records. The research team in Bangladesh also conducted a screening of all households for identifying individuals with fever for more than 2 weeks, splenomegaly and positive rK39 test in one randomly selected camp catchment area (6 villages) where a camp had been held earlier to determine the sensitivity of the camp search in identifying new VL cases existing in the community. Furthermore, new VL cases identified by the house to house screening but who had not participated in the camp were also interviewed to seek reasons for not participating in the camp held earlier.

The program cost for implementing the camp and index case search strategies was estimated using an ingredients costing method. Costing ingredients included costs for training, development and production of training materials, rK39 kits, per diem costs and costs for transport, communication and materials.

### Statistical methods

Data was entered and managed centrally using EPI Info software. Simple descriptive (means and proportions) summary statistics and tests of significance for difference in means and proportions were estimated. Sensitivity of the camp in Bangladesh was calculated as the percentage of new VL cases identified by the camp out of the total of new cases identified by the camp and the additional new cases identified in the house to house search.

## Results

### Performance and feasibility

Altogether 48 camps (Bangladesh-27, India-19 and Nepal-2) and 81 index case searches (India-36, Nepal-45) were conducted by the health services across 50 health center areas (Bangladesh-4 Upazillas, India-9 PHCs and Nepal-37 VDCs). Patients with fever for more than 2 weeks or known VL cases with skin lesions attending camps varied (mean - 12.9, range - 1 to 50 in Bangladesh; mean - 11.8, range - 2 to 30 in India; and mean - 29.5, range - 24 to 35 in Nepal). Male attendance at camps (range 48 to 56%) was similar across the 3 countries (Table 1). Camp attendees were younger by 5 years in Bangladesh (mean age 27.4 years) compared to India and Nepal (P-value <0.0001). Of the 225 camp attendees, 193 were recorded to have fever plus splenomegaly of whom 5 (2.6%) tested positive for rK39 test in India compared to 22% of patients with fever and splenomegaly tested positive for rk39 test in Bangladesh. On the other hand, in Nepal, 3 of 59 (5%) patients with fever for more than 2 weeks had splenomegaly, all of whom were positive for rK39 test. The camp search strategy yielded 40 new VL cases in Bangladesh (mean 1.5 new VL case per camp) compared with 5 (mean 0.3) and 3 (mean 1.5) new VL cases in India and Nepal respectively. Additionally in Bangladesh, 14 new PKDL cases were identified in the camps compared to one in India and none in Nepal. All newly identified cases in India and Nepal completed treatment. In Bangladesh, 13 of 40 (32.5%) VL cases completed their treatment (Table 1). Treatment completion could not be ascertained for the remaining 27 VL cases and all of the 14 PKDL cases in Bangladesh as they were lost to follow-up. In the single camp catchment area (6 villages) in Bangladesh where the camp was followed by screening of all households in the village, 13 new cases (6 new VL and 7 new PKDL cases) were identified in addition to 9 new cases (3 new VL and 6 new PKDL cases) identified earlier in the camp giving an overall sensitivity of 41% (95% CI, 21% – 63%) for the camp search strategy when compared with the house to house screening strategy (results not shown). None of the 13 new cases identified subsequently in the household screening had received information about the camp. Qualitative assessment (structured observations) of the camp search strategy showed adequate preparatory activities (training, scheduling, selection of location etc.) except in India where information education and communication (IEC) activities in the community were inadequate to promote attendance at camps. Screening of patients, spleen examination and counseling for rK39 tests were correctly done at camps. However one of the two camps in Nepal did not have an adequate supply of rK39 kits for testing. It was also noted that patient and camp records were inadequately maintained in 10 of 19 camps in India whereas in Bangladesh the results of spleen examination were not recorded correctly. Clinical skills of medical officers in spleen examination in India were observed to be inadequate as they tended to incorrectly record splenic enlargement. District/National program managers in group discussions listed delayed budgetary sanctions, inadequate time and resources to prepare for camp activities, inadequate training and supervision as major challenges in implementing the camp search strategy.

**Table 1:** Camp search intervention carried out by health services in Bangladesh, India and Nepal

	**Bangladesh**	**India**	**Nepal**
No. of camps held	27	19	2
Mean no. of camp attendees^$ ^(range)	12.9 (01 – 50)	11.8 (02 – 30)	29.5 (24 – 35)
% (No.) male	48% (168/349)	52.9% (119/225)	56% (33/59)
Mean (SD) age in years	27.4 (19.1)	33.1 (18.5)	33.8 (20.3)
Detection of VL			
*- with splenomegaly*	52% (183/349)	85.8% (193/225)	5% (3/59)
*- rK39 test done*	100% (183/183)	100% (193/193)	100% (3/3)
*- rK39 positive*	22% (40/183)	2.6% (5/193)	100% (3/3)
*- referred for treatment*	100% (40/40)	100% (5/5)	100% (3/3)
*- completed treatment*	33% (13/40)	100% (5/5)	100% (3/3)
*- No. of new VL cases detected*	40	5	3
*- No. of new VL cases detected per camp*	1.5	0.3	1.5
Detection of PKDL			
*- with suspected skin lesions*	5% (17/349)	2.2% (5/225)	0% (0/59)
*- rK39 test done*	100% (17/17)	100% (5/5)	--
*- rK39 test positive*	82% (14/17)	20% (1/5)	--
*- referred for treatment*	100% (14/14)	100% (1/1)	--
*- completed treatment*	0% (0/14)	100% (1/1)	--
*- No. of new PKDL cases detected*	14	1	0
*- No. of new PKDL cases detected per camp*	0.5	.05	0
No. of new cases (VL + PKDL) detected	54	6	3
No. of new cases detected per camp	2.0	0.32	1.5

^$^Camp attendees with fever for more than 2 weeks or past case of VL with skin lesions.

In Nepal, the index case search strategy conducted for 45 index VL cases did not yield any new VL case while a similar search around 36 index cases in India yielded 3 new VL cases (average 0.08 new VL case per index case search) and 1 new PKDL case (average 0.03 new PKDL case per index case search) (Table 2). All newly identified VL and PKDL cases in India initiated and completed treatment. A qualitative assessment of the index case search strategy showed that health workers in Nepal though trained did not perform a spleen examination or rK39 test during household screening and instead referred a suspected patient with fever for more than 2 weeks to the health center for further management.

**Table 2:** Index case search intervention carried out by health services in India and Nepal (Index case search was not implemented by Bangladesh)

	**India**	**Nepal**
No. of index cases identified	36	45
Total no. of households (population) screened	1627 (9470)	1046 (7091)
Detection of VL		
*- fever >2 weeks*	0.3% (29/9470)	0% (0/7091)
*- rK39 test done*	100% (29/29)	--
*- rK39 test positive*	10% (03/29)	--
*- referred for treatment*	100% (3/3)	--
*- completed treatment*	100% (3/3)	--
*- No. of new VL cases detected*	3	0
*- No. of new VL cases detected per index case search*	.08	0
Detection of PKDL		
*- with suspected skin lesions*	0.01% (1/9470)	0% (0/7091)
*- rK39 test done*	100% (1/1)	--
*- rK39 test positive*	100% (1/1)	--
*- referred for treatment*	100% (1/1)	--
*- completed treatment*	100% (1/1)	--
*- No. of new PKDL cases detected*	1	0
*- No. of new PKDL cases detected per index case search*	.03	0

### Costs

Table 3 shows the costs related to camp search strategy for detection of VL and PKDL cases. The cost per camp was US$ 283, US$ 349 and US$ 688 in Bangladesh, India and Nepal respectively. Cost per detection of new case was highest in India (US$ 1046) followed by Nepal (US$ 459) and Bangladesh (US$ 105). Training start-up costs accounted for 57% of total costs in Nepal, 69% in India and 79% of total costs in Bangladesh. The cost per detection of new case was lower (Nepal – US$ 199, India – US$ 320, Bangladesh – US$ 22) if training costs were excluded. Costs for IEC activities varied between 1 to 3% of total camp search strategy costs.

**Table 3:** Costs equivalent to USD in 2010 for camp search strategy in Bangladesh, India and Nepal (Figures in parenthesis are% of total cost)

	**Bangladesh (USD)**	**India**^**$**^**(USD)**	**Nepal (USD)**
Training	4448 (79%)	2177 (69%)	778 (57%)
Communication & social mobilization	191 (3%)	59 (2%)	23 (1%)
Camp day activities	1022 (18%)	902 (29%)	575 (42%)
Total cost	5661	3138	1376
- cost per camp	283	349	688
- cost per new case detected	105	1046	459
- cost per camp (excl. training costs)	61	107	299
- cost per new case detected (excl. training cost)	22	320	199

^**$ **^Nine out of 19 camps where 3 new cases were identified are included in the cost analysis.

The cost per new case detected by the index case search strategy was US$ 1311 of which transport accounted for 62% and personnel (per diems) accounted for 32%. Training costs accounted for 4% of total costs (Table 4). No new VL or PKDL case was detected in Nepal using this strategy but the cost was about US$ 4 per household searched in Nepal. The absolute cost of index search was US$ 97 and US$ 91 in India and Nepal respectively (Table 4).

**Table 4:** Costs equivalent to USD in 2010 for index case search strategy in India and Nepal

	**India**^**$**^**(USD)**	**Nepal(USD)**
Training	109 (4%)	259 (6%)
Personnel	832 (32%)	2656 (65%)
Transport	1630 (62%)	1202 (29%)
rK39 kits	47 (2%)	0 (0%)
Total cost	2622	4117
- cost per new case detected	1311	--
- cost per household searched	3	4
-cost per index case search	97	91

Figures in parenthesis are% of total cost (Bangladesh did not implement index case strategy).

^**$ **^Twenty-seven out of 36 index case search where 2 new cases were identified from 950 households are included in the cost analysis.

## Discussion

Our study highlights challenges faced by national programs for VL elimination in adapting ACD strategies for early case detection and treatment in Bangladesh, India and Nepal. Overall, the camp search strategy was superior to the index search strategy in terms of costs as well as yield comparable with earlier evidence [11]. Camps conducted by health services in Bangladesh showed lower sensitivity compared to those conducted by researchers in an earlier study [11]. The number of new cases identified through the camp strategy could have been up to 60% higher especially in Bangladesh if camp awareness and promotional activities were done adequately. This was also reflected in practice where less than 3% of the total program expenditure for implementing the ACD strategy was spent on IEC and related activities. Spleen examination by a medical officer in a camp setting or by a health worker at the household level was another challenge. A large proportion of patients (95% in India, 30% in Bangladesh) with fever for more than 2 weeks and diagnosed with splenomegaly had a negative rK39 test. This was most likely due to poor skills of the medical officers in clinical examination for spleen size as observed by the researchers during the camp especially in India. This subjected a large proportion of patients to unnecessary testing with rK39. On the other hand, an alternative cause for chronic fever and splenomegaly with a negative rK39 test cannot be ruled out. Another major challenge seen in Bangladesh was the high proportion (two-thirds) of newly identified patients who did not avail of the referral and initiate and complete treatment. Interestingly, the cost of detection of a new case was substantially higher in India compared to Nepal and Bangladesh and also compared to an earlier study where ACD strategies were implemented directly by researchers in collaboration with the health services [11]. The higher cost could be due to the larger area (complete district) covered by the health services, the higher number of staff involved in the camps, the lower number of VL cases detected and some other contextual costs. Lastly, our study continues to document the large burden of PKDL in Bangladesh seen in earlier studies [9,11].

Our study had a few limitations. The index case search strategy could not be evaluated in Bangladesh as the health system had not yet adopted it as a strategy into their program. The evaluation of the camp search strategy in Bangladesh was based on retrospective interviewing of the camp team and camp attendees rather than based on direct observations of activities before and during the camp.

## Conclusions

Our study concludes that national programs can adapt ACD strategies for detection of more VL/PKDL cases. However adequate time and resources are required for training, planning and strengthening referral services to overcome challenges faced by the programs in conducting ACD.

## Abbreviations

VL: Visceral leishmaniasis; PKDL: Post-kala-azar dermal leishmaniasis; NKEP: National program for kala-azar elimination; ACD: Active case detection; RTAG: Regional technical advisory group; PHC: Primary health center; VDC: Village development council; CI: Confidence interval; IEC: Information education and communication.

## Competing interests

The authors declare that they have no competing interests.

## Authors’ contributions

MMH, SH, NAS, PM, MRB, PD, SK, CHG, SR, BA, AK, EN and DM design and implemented the study. MMH, SH, AK, and DM wrote the paper; MMH & EN performed the data analysis. All authors read and approved the final manuscript.

## Pre-publication history

The pre-publication history for this paper can be accessed here:

http://www.biomedcentral.com/1471-2458/12/1001/prepub
